# The E/e’ ratio difference between subjects with type 2 diabetes and controls. A meta-analysis of clinical studies

**DOI:** 10.1371/journal.pone.0209794

**Published:** 2018-12-27

**Authors:** Giacomo Zoppini, Corinna Bergamini, Alessandro Mantovani, Marco Dauriz, Giovanni Targher, Andrea Rossi, Enzo Bonora

**Affiliations:** 1 Section of Endocrinology, Diabetes and Metabolism, Department of Medicine, Azienda Ospedaliera Universitaria Integrata, University of Verona, Verona, Italy; 2 Section of Cardiology, Department of Medicine, University and Azienda Ospedaliera Universitaria Integrata of Verona, Verona, Italy; Universita degli Studi di Roma La Sapienza, ITALY

## Abstract

Type 2 diabetes is associated with an increased risk of heart failure. Left ventricular diastolic dysfunction and type 2 diabetes are frequently associated. Using echocardiography, we know that tissue Doppler imaging E/e’ ratio is a reliable predictor of left ventricular filling pressure. We performed a systematic review and meta-analysis to investigate the averaged E/e’ ratio value in patients with type 2 diabetes compared to non-diabetic controls. In the analysis we included cross-sectional studies providing the averaged E/e’ ratio. Subgroup/sensitivity analyses were conducted according to variables known to influence E/e’ ratio measurements. The analysis included 15 cross sectional studies with 877 type 2 diabetes patients and 1193 controls. The weighted mean difference showed higher values in diabetes (WMD 2.02; 95% CI 1.35, 2.70; p<0.001). The result was consistent in the subgroup/sensitivity analyses. Visual inspection of the funnel plot did not identify substantial asymmetry and the Egger test for funnel plot asymmetry showed a p value of 0.36. In conclusion, our assessment suggests that averaged E/e’ ratio is consistently increased in patients with type 2 diabetes compared to non-diabetic controls in the absence of cardiovascular diseases and complicated hypertension. This alteration may be a precocious diastolic alteration in the diabetic cardiomyopathy.

## Introduction

Diastolic dysfunction is an important cause of heart failure (HF) with preserved ejection fraction (pEF) in diabetes, overall in type 2 diabetes [1]. Considering the worldwide epidemic increase in type 2 diabetes incidence along with complications [2], it is presumable that this cardiac condition will become a major public health burden [3]. Epidemiologic studies have shown that different grades of diastolic dysfunction may be detected in patients with type 2 diabetes [4–6].

Left ventricular (LV) end-diastolic pressure (LVEDP) or pulmonary capillary wedge pressure (PCWP) are frequent measures used to assess LV diastolic function [7]. In this respect, echocardiography is the mainstay for the noninvasive evaluation of diastolic function [7]. Early mitral annular velocity (e’) obtained by tissue doppler imaging estimates LV myocardial relaxation activity: e’ less than 10 (lateral annular location) and e’ less than 7 cm/sec (septal annular location) may suggest impaired myocardial relaxation [7].

With mitral early filling velocity E, the ratio E/e’ is largely used to estimate the left ventricular filling pressure (LVFP) and its use is recommended by the echocardiographic Societies to evaluate diastolic function and HFpEF [7–8].

Despite a large use of E/e’ ratio, the extent of its alteration in type 2 diabetes without cardiovascular complications is still elusive.

In the present meta-analysis we summarize the averaged E/e’ ratio mean difference between subjects affected by type2 diabetes without cardiovascular complications and control subjects. We also summarize the averaged E/e’ ratio mean difference in various clinical conditions in patients with type 2 diabetes that may confound this relationship.

## Materials and methods

We conducted this systematic review and meta-analysis in accordance with the PRISMA guidelines [9] and registered our project with the international prospective register of systematic reviews (PROSPERO—number CRD42018093585)

### Search strategy

Four investigators (G.Z., A.M., M.D., G.T.) independently searched PubMed, Web of Science and Scopus for pertinent articles. Furthermore, the investigators scanned references of retrieved articles and pertinent reviews to detect further studies.

**Fig 1:**
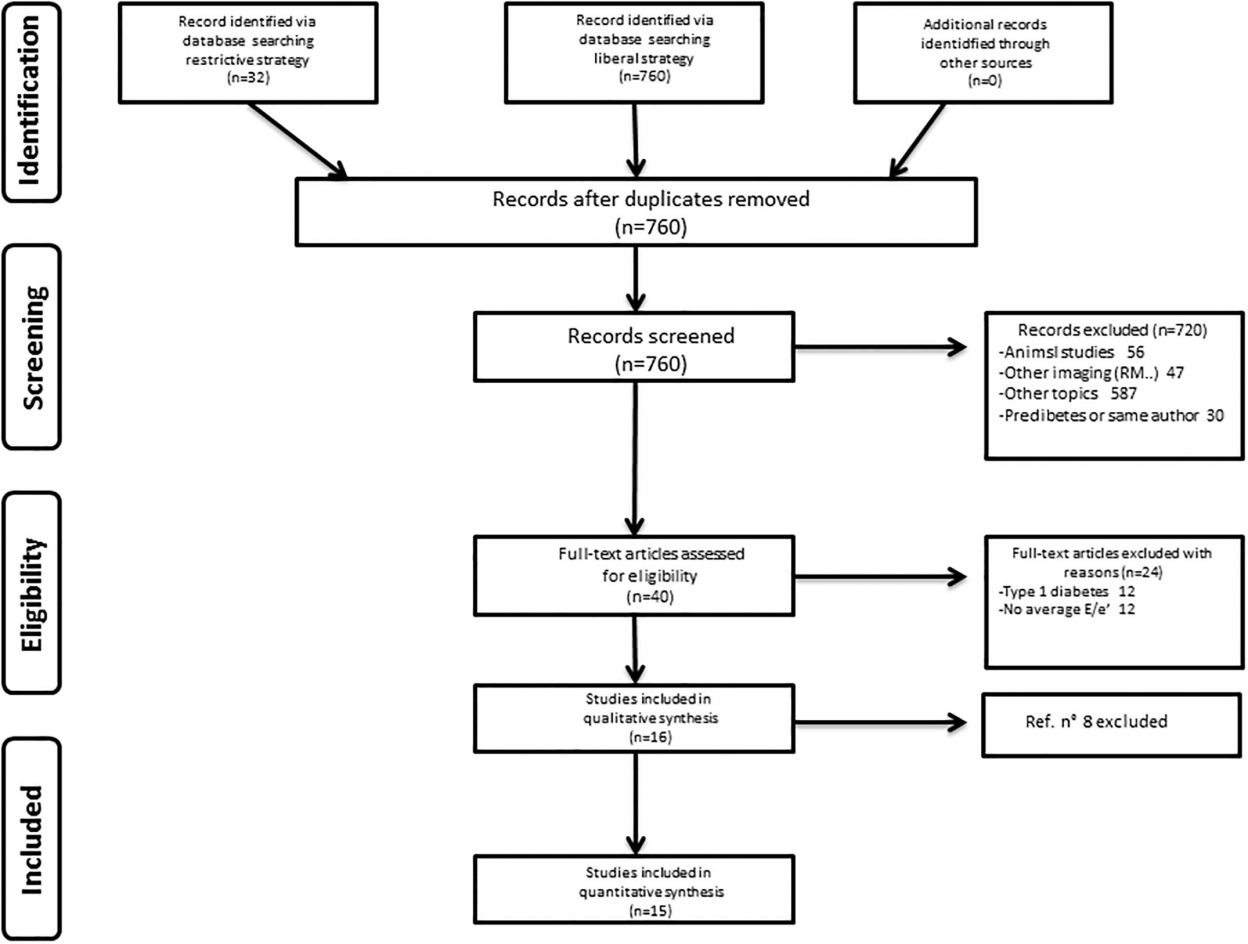
The PRISMA flowchart of the systematic search and quantitative synthesis.

As reported in Fig 1, we performed two kinds of researches: 1) more liberal using generic items (‘Diabetes’, ‘Diastolic dysfunction’, ‘Controls’) and (‘Diabetes’, ‘Tissue doppler’, ‘Controls’) that retrieved 760 studies; 2) using more restrictive items: (‘Diabetes’, ‘Tissue Doppler velocity’, ‘Controls’) and (‘Diabetes’, ‘e/e’, ‘Controls’) that retrieved 32 studies.

The PubMed search was carried out by using isolated terms, not phrases nor Boolean operators in order to retrieved the larger number of references (free word searching). The terms were written directly in the search mask. Limits: only articles published in English were considered, we only included studies reporting data obtained by transthoracic echocardiography. Duplicates were manually searched. The last search update was on January 20, 2018.

### Eligibility criteria and identification of study

Definition and diagnosis of diabetes has been the same since 2010 [10]. Studies were included if they provided the mean E/e’ ratio (averaged TDI e’ values of lateral and septal annular region), comparing values in type 2 diabetes patients and in control subjects. Inclusion criteria were cross-sectional studies reporting the mean E/e’ ratio in adult patients with type 2 diabetes without any previous cardiovascular diseases except non complicated hypertension and matched or unmatched controls subjects. Exclusion criteria were studies reporting either septal or lateral annular e’ measures, studies on those under 18 years and studies on subjects with known cardiovascular diseases, including atrial fibrillation.

### Study selection and data extraction

The four authors reviewed the findings of the electronic search and selected the articles potentially relevant to the topic of interest. The identified articles were downloaded and then assessed against the eligibility criteria. Any discrepancy in an author’s opinion on the inclusion of an article was resolved by consensus and/or by involving the other authors (C.B., A.R., E.B.). Two reviewers (G.Z., M.D.) independently extracted the data from each study, which were recorded into a pre-defined collection sheet. Data extracted from each study included the number of type 2 diabetes patients and controls, the mean of averaged E/e’ ratio of both groups and the standard deviations of both groups along with other data (Table 1).

**Table 1:** Characteristics of enrolled case-control studies of averaged E/e’ ratio in type 2 diabetes patients compared to non-diabetic controls. OP: outpatient; CP: consecutive patient; H: healthy control. NOS: Newcastle-Ottawa scale. Case: type 2 diabetic patients; Control: healthy non-diabetic controls.

		Case					Control								
Study	Country	Source	n	Age yrs	Sex M/F	E/e'	Source	n	Age yrs	Sex M/F	E/e'	Matching	NOS score	Hypertension	HbA1c
Tayebjee MN	UK	OP	54.00	68±5	43/12	10.9±1.3	H	31.00	66±5	18/13	8.1±2	age and sex	6	1	7.3
Govind SC.	Sweden	CP	31.00	49.2±6.3	20/11	10.8±2.4	H	13.00	49.7±5.4	8/5	7.9±0.7	age. LV size and ECG parameters	6	0	8.1
Yazici M.	Turkey	OP	72.00	49.1±9.8	36/36	6.2±3.8	H	50.00	46.1±9.8	17/33	6.2±2.8	no	6	0	8.3
Mogelvang R.	Denmark	OP	65.00	68±11	42/13	12.7±1.5	NA	533.00	51±14	233/300	9±1.3	no	6	0	
Andersson CH.	Denmark	OP	31.00	58±12	16/15	9.9±5.8	H	31.00	58±12	16/15	7.0±1.6	age.sex.hypertension	8	1	
Tayyareci Y.	Turkey	CP	60.00	58.2±11.3	21/39	8±1.6	H	40.00	57.4±8.1	12/28	4.8±1.4	age and sex	7	0	7.6
Ernande L.	France	OP	114.00	52±4.5	60/45	10.9±3.6	H	88.00	51.7±2.6	30/58	7.7±1.7	age and sex	7	1	7.7
Ceyhan K.	Turkey	CP	48.00	56±11	28/20	11.5±3.0	H	60.00	56±11	32/28	9.8±2.2	age and sex	7	0	7.8
çiftel S.	Turkey	CP	21.00	54.1±5.7	11/10	4.9±1.9	H	40.00	53±6.8	17/23	5.6±1.9	no	5	0	9.4
Conte L.	Italy	OP	44.00	60.9±6.6	23/21	9.3±3.4	H	24.00	58.4±9.4	13/11	7±1.6	no	4	1	7.3
Erdogan D.	Turkey	NA	45.00	51.6±7.2	19/26	10.25±3.11	NA	43.00	50.4±8.5	18/25	9.05±2.41	no	5	1	7.4
Atas J.	Turkey	CP	40.00	50.5±7.3		7.7±2.3	H	40.00	48.4±6.7		6.2±1.3	age and sex	7	0	7.3
Bakirci EM.	Turkey	CP	132.00	54.5±9.6	76/56	8.9±2.8	H	80.00	53.2±9.0	50/30	8.6±2.5	age and sex	7	0	8.4
Loncarevic B	Serbia	CP	70.00	54.8±7.7	38/32	10.11±3.27	H	80.00	54.8±4.9	44/36	7.40±1.42	age and sex	6	0	6.7
Vukomovic V.	Serbia	CP	50.00	55±7	26/24	9.4±3	H	40.00	50±9	12/18	7.0±1.8	no	5	0	7.3

### Quality assessment of study design

Methodological quality of selected cross-sectional studies was estimated using the Newcastle-Ottawa scale (NOS). The NOS explores risk of bias in three different domains: selection, comparability and outcome/exposure. A maximum cumulative score of 9 (stars) points can be obtained: four stars for selection, two stars for comparability and three stars for outcome/exposure. Studies were classified as high-risk (1–3 points), intermediate (4–5 points) or low-risk of bias (6–9 points) [11]. Three authors made the NOS score independently and a final agreement was reached (S2 Table). The authors’judgments about each domain of the Newcastle-Ottawa Scale is presented in S3 Table, while the Cochrane Risk of Bias Study-by-Study in S4 Table.

### Analysis

The analysis investigated the differences between averaged E/e’ ratio between patients with type 2 diabetes and non-diabetic controls. We further conducted a subgroup/sensitivity analyses to explore the possible sources of heterogeneity.

### Statistical analysis

Mean values and standard deviation (SD) of the variables of interest were collected for the analysis. If data were reported only as median and interquartile range (none of the final studies included), published and online Cochrane’s recommendations to approximate the values of mean and SD can be followed [12]. One study reported the geometric mean, however as the measure of interest was the difference of the means, we included this difference measure in the analysis.

The weighted mean differences (WMDs) were used to compare the averaged E/e’ ratio between the case and control subjects. The pooled data were calculated by using a random-effect model to achieve a more conservative assessment. Statistical heterogeneity was estimated using Cochrane’s Q test and the I^2^ statistics. Heterogeneity was likely if Q>df (degree of freedom), and confirmed if P ≤ 0.10. Quantification of heterogeneity was performed by using I^2^ statistics. The degree of heterogeneity was defined as none, low, moderate or high according to I^2^ values of 0–24.9%, 25–49.9%, 50–74.9% and > 75%, respectively. Publication bias was qualitatively assessed by the visual inspection of funnel plot asymmetry of the MD against their standard errors. The Egger’s regression asymmetry was also calculated and a P <0.05 was considered to be suggestive of a statistically significant publication bias. Meta-analysis was performed with R metaphor.

This study was conducted in compliance with the Cochrane Collaboration and the Preferred Reporting Items for Systematic Reviews and Meta-Analyses guidelines [13–14]. A p value less than 0.05 was considered as statistically significant.

## Results

The literature search produced 32 titles for the restrictive search strategy and 760 for the more liberal search approach. No additional articles were found by an independent search. All articles were screened yielding 40 studies as potentially relevant and full-text was retrieved. Twenty four studies were excluded: thus the sixteen remaining papers were selected for the qualitative synthesis, while fifteen were selected for the quantitative synthesis. The search on Embase and Scopus did not add further evidence to the Medline findings.

Therefore, 15 studies included in the meta-analysis provided transthoracic echocardiographic data on TDI values, in particular they provided results on averaged E/e’ ratio.

The PRISMA flowchart of our systematic search and quantitative synthesis is reported in Fig 1. The characteristics of the studies included are summarized in Table 1 [15–29].

**Fig 2:**
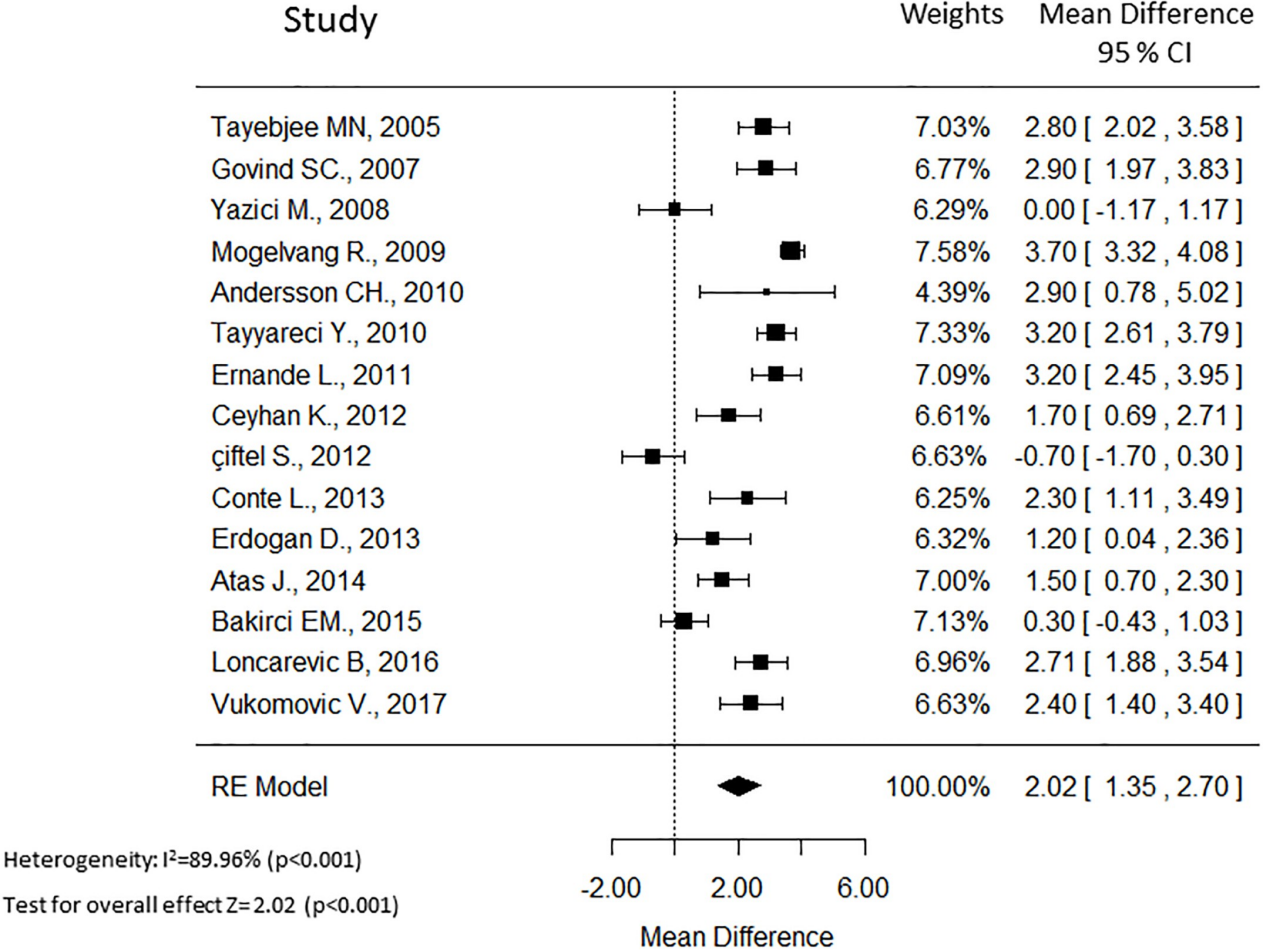
The Forest plot of the weighted mean difference (WMD) of E/e’ ratio with 95% C.I. of the included studies that compared averaged E/e’ between patients with and without diabetes. A positive value signifies that E/e’ is higher in patients with diabetes.

**Fig 3:**
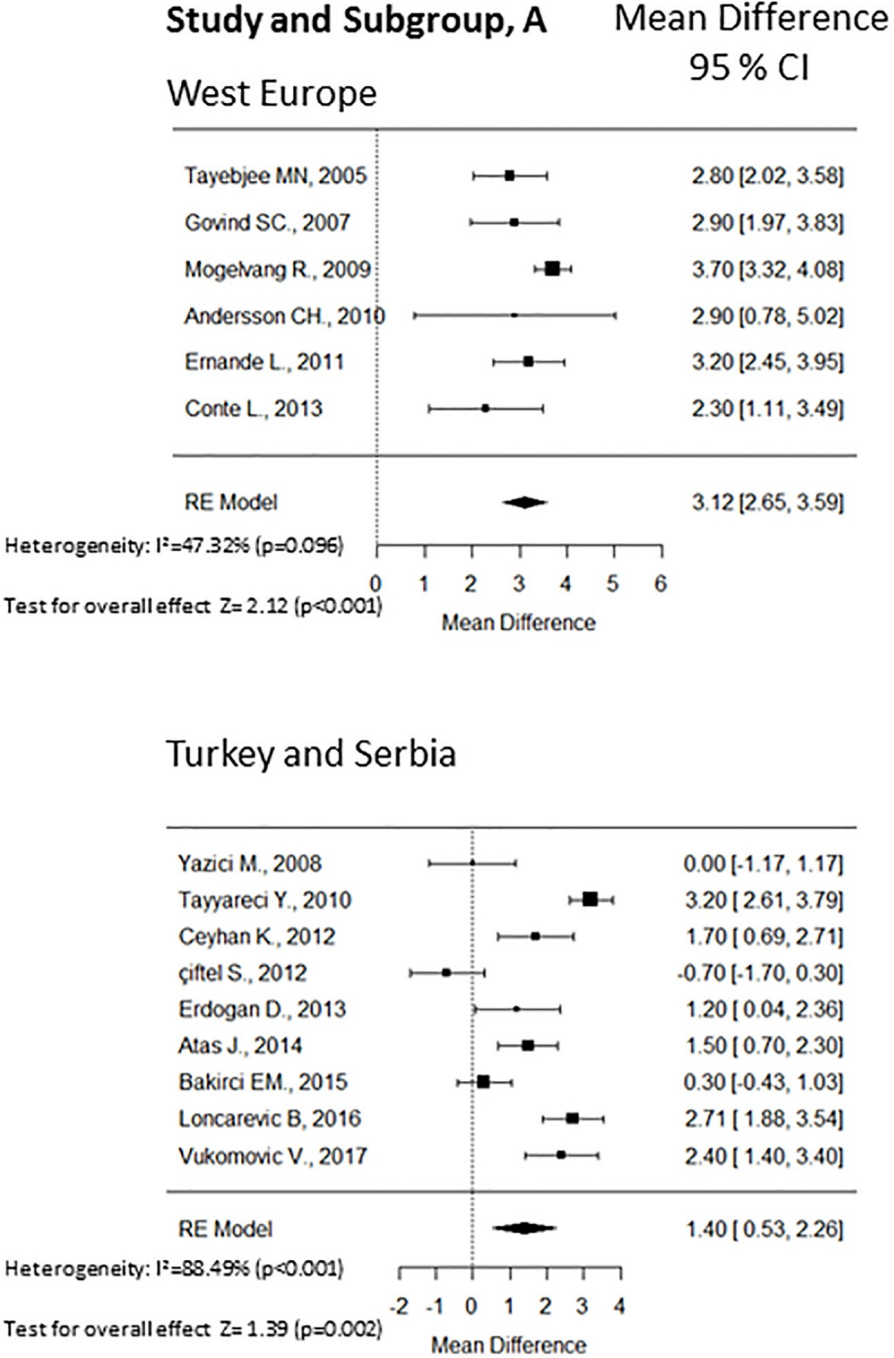
The Forest plot of the WMD with 95% C.I. of the subgroup analyses: Panel A, region; panel B, matching; panel C, hypertension; panel D, glycemic control. A positive value signifies that E/e’ is higher in patients with diabetes.

Fifteen cross sectional observational studies provided values of the averaged E/e’ ratio between patients with type 2 diabetes (n = 877) and non-diabetic controls (n = 1193). The WMDs forest plot of this analysis is shown in Fig 2. Overall, type 2 diabetes patients without cardiovascular diseases exhibited a significantly higher averaged E/e’ ratio (WMD 2.02; 95% CI 1.35, 2.70; p<0.001, Fig 2) with high heterogeneity (I^2^ = 89.9%; p < 0.001). Considering the significant heterogeneity among the studies, we conducted subgroups/sensitivity analyses. The region, the selection of controls (matching vs not matching), presence of non-complicated hypertension, and glycemic control (HbA1c ≤ 7.3% vs HbA1c > 7.3%) may have influenced the summary combination, therefore we performed subgroups analyses according to these factors. The weighted forest plots of these analyses are shown in Fig 3. According to the region, the studies were divided in two subgroups and higher heterogeneity was found in studies from Serbia and Turkey (I^2^ = 88.5%; p < 0.001). The overall WMD was 3.12 (95% CI 2.65, 3.59) in the west European region and 1.40 (95% CI 0.53, 2.16) in Serbia and Turkey region. High heterogeneity was found independently of controls matching. The not matching subgroup presented I^2^ = 92.7% (p < 0.001) while the matching subgroup had I^2^ = 83.0% (p < 0.001). The presence of non-complicated hypertension was associated with a lower heterogeneity (I^2^ = 56.2%; p = 0.072) compared to the absence of hypertension (I^2^ = 93.2%; p < 0.001). Moreover, the WMD was 2.52 (95% CI 1.79, 3.24) when non-complicated hypertension was present and 1.81 (95% CI 0.88, 2.73) in the absence of hypertension.

Substantial low heterogeneity was found in the subgroup of studies with low mean HbA1c (≤ 7.3%) with I^2^ = 39.7% (p < 0.177) respect to the studies with higher mean HbA1c (> 7.3%) with I^2^ = 91.8% (p < 0.001). The HbA1c was chosen because it was the median value of the means. The WMD was 2.34 (95% CI 1.82, 2.85) in the subgroup with better glycemic control and 1.50 (95% CI 0.43, 2.57) in the worse glycemic control subgroup. S1 Table shows the subgroups/sensitivity analyses respect to the number of participants to each study and to the NOS score. The overall effect was more stable when studies included more than 45 subjects and the NOS score was above 6.

**Fig 4:**
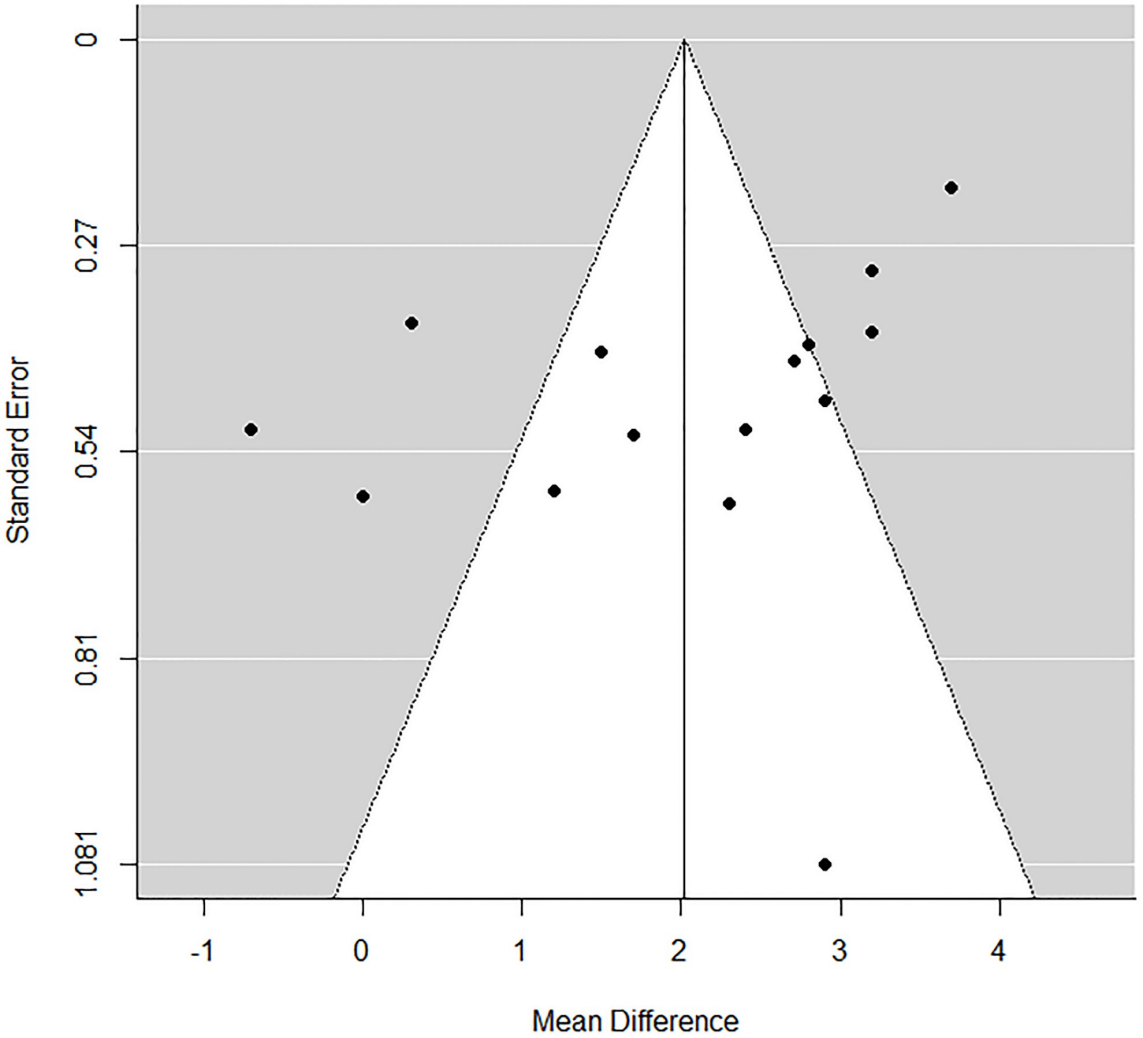
The Funnel plot analysis of the studies included in the analysis.

None of the studies ranked between 1–3 NOS score, four studies (26%) were between 4–5 while the majority of studies were above 6 NOS score. Visual inspection of the funnel plot (Fig 4) did not identify substantial asymmetry and the Egger test for funnel plot asymmetry showed a p value of 0.36.

## Discussion

Our meta-analysis investigated the WMD of averaged E/e’ ratio between patients with type 2 diabetes without cardiovascular complications and non-diabetic controls. Care was taken in selecting studies that clearly reported that included subjects were free of cardiovascular complications, but non complicated hypertension.

We found a significantly higher averaged E/e’ ratio in patients with type 2 diabetes. These findings may suggest the presence of LVDD in type 2 diabetes patients in the absence of significant cardiovascular complications and they were consistent either in subjects with and without non complicated hypertension or good and bad glycemic control. Thus, the results of this meta-analysis seems to indicate a possible direct detrimental effect of T2DM on the diastolic performance of myocardium.

Diastolic alterations may be a precocious phenomenon of the diabetic heart: indicated as diabetic cardiomyopathy [30].

Heart failure, especially HFpEF, and type 2 diabetes are frequently found associated in the same patient [31–32]. The coexistence of the two diseases is associated with a more severe clinical status and the prognosis is encumbered by an increased risk of all-cause and cardiovascular mortality [33]. We excluded subjects with cardiovascular diseases, that are responsible for most of the case of HF in diabetes. The other main cause of HF is arterial hypertension, that in our study was considered in the subgroups/sensitivity analyses. Therefore, after the exclusion of the main causes, HF may be the consequence of T2DM related-processes [34]. Major mechanisms of myocardial alteration in T2DM are insulin resistance/hyperinsulinemia and pre-diabetic conditions, such as obesity, dysglycemia and others. Hyperinsulinemia and dysglycemia may be present years or even decades before overt diabetes, likely contributing to myocardial dysfunction during this period [35]. In fact, left ventricular diastolic dysfunction may be detected in as many as about 75% of T2DM patients. Moreover, according to demographic characteristics of these patients, that may include younger age, normal blood pressure and optimal glycemic control, it can be supposed that left ventricular dysfunction may develop early in the course of the disease [1, 36–37].

Numerous metabolic abnormalities, commonly found in diabetes, may be detrimental to left ventricular diastolic function. Among these metabolic abnormalities are to be underlined for their importance nonenzymatic glycation of proteins, lipotoxicity and microvascular rarefication: all these abnormalities eventually lead to apoptosis and fibrosis [34].

One simple clinical approach to detect myocardial dysfunction is transthoracic echocardiography with TDI investigation. In particular, the E/e’ ratio, that estimates the left ventricular filling pressure, is one of the most important parameters to detect diastolic dysfunction in subjects with pEF. When E/e’ ratio increased at rest, it associates with adverse outcomes [38].

E/e’ ratio has been shown to possess a good prognostic impact on different outcomes such as all-cause mortality, cardiovascular death and heart failure hospitalization in various studies [39–40]. Furthermore, a 4-year longitudinal study showed that progressive worsening in E/e’ ratio was associated with an increased incidence of heart failure [41]. We believed that the novelty of our study are: the care of the selected patients thus proving that E/e’ may represent an early alteration, and second that patients with normal blood pressure and higher level of HbA1c showed a higher variability in the estimates. Therefore, even patients in good metabolic control may develop alteration in E/e’ ratio and hypertension may contribute to this alteration. The results of our meta-analysis are clinically relevant as they indicate that a single a reproducible parameter may be precociously altered. However, it should be remembered that we do not have cutoff levels of averaged E/e’ under the value of 14. Future studies are needed to evaluate the linear prognostic value of this parameter.

An advance in our knowledge is the routine measure of E/e’ ratio, as marker of increased LV filling pressure, given the high prevalence of hypertension and heart failure in T2DM. Screening HF is important since its two early phases, stage A (HF risk factors), and stage B (characterized by structural or functional evidence of myocardial disease), are asymptomatic [42]. It is of note that in these two HF stages T2DM is cited and therapy, with protective effect, has an established indication in the prevention of incident HF. Thus, echocardiography is a test that may potentially influence therapeutic decision making.

### Sources of heterogeneity

In the present meta-analysis there was a substantial heterogeneity among the publications (I^2^ > 50%). Several factors might explain the heterogeneity, such as the characteristics of both diabetes and controls populations. Another possible factor of heterogeneity is the different echocardiographic equipment in the diverse centers. Moreover, different factors, such as age, glycemic control, hypertension may be associated with variations of E/e’ ratio, for this reason we performed subgroups analyses to take into account these possible confounders. As shown in Fig 2, region, glycemic control and hypertension can decrease the heterogeneity.

### Study strengths and limitations

The major strength of this study is the comprehensiveness of the literature retrieval and review. All studies included subjects with no cardiovascular diseases. We also included important clinical factors such as glycemic control and hypertension. The data of the studies are as complete as possible, and we included only case-control studies with a fair representation of recent publications. Moreover, we performed subgroups analyses to further illustrate the result of this topic. And finally, as far as we know the present is the most comprehensive and updated synthesis of E/e’ ratio in patients with type 2 diabetes.

This study has limitations. The matching between cases and controls was not consistent in all studies. The inclusion and exclusion criteria slightly differed among studies. The clinical characteristics of T2DM patients were not complete in some study. Diabetes duration was not reported in many studies. Heterogeneity is substantial among studies even in the subgroup analyses. Despite of all the limitations, the results of this analysis are consistent.

## Conclusions

In conclusion, our assessment suggests that averaged E/e’ ratio is consistently increased in type 2 diabetes patients compared to non-diabetic controls in the absence of cardiovascular diseases and complicated hypertension. This alteration may be a precocious diastolic alteration of the diabetic cardiomyopathy. Nevertheless, the prognostic role of E/e’ should be considered with caution. Future studies relating outcomes to E/e’ in diabetes may clarify the real prognostic importance of this parameter.

## Supporting information

S1 Table PRISMA checklist. (DOC)

S2 Table Supplementary. Subgroups/Sensitivity analyses of the overall effect of studies with ≤ 45 and > 45 participants; studies with NOS > 6 and ≥ 6 score.
(DOCX)

S3 Table Newcastle-Ottawa quality assessment scale (NOS). (DOCX)

S4 Table Risk of bias graph: Review authors’judgments about each domain of the Newcastle-Ottawa scale presented as percentages across all considered studied. (PDF)

S5 Table Cochrane risk of bias stusy-by-study table. (PDF)

## Abbreviations

HbA1c: glycated hemoglobin
HF: heart failure
LV: left ventricle
LVDD: left ventricular diastolic dysfunction
LVEDP: left ventricular end-diastolic pressure
LVEDV: left ventricular end diastolic volume
LVEF: left ventricular ejection fraction
LVFP: left ventricular filling pressure
NOS: Newcastle-Ottawa scale
PCWP: pulmonary capillary wedge pressure
pEF: preserved ejection fraction
T2DM: type 2 diabetes mellitus
TDI: tissue Doppler imaging
WMD: weighted mean difference

## References

[1] Seferović PM, Petrie MC, Filippatos GS, Anker SD, Rosano G, Bauersachs J, et al Type 2 diabetes mellitus and heart failure: a position statement from the Heart Failure Association of the European Society of Cardiology. Eur J Heart Fail 2018 3 8 10.1002/ejhf.1170 [Epub ahead of print] Review. 29520964

[2] Koye DN, Magliano DJ, Nelson RG, Pavkov ME. The Global Epidemiology of Diabetes and Kidney Disease. Adv Chronic Kidney Dis 2018; 25: 121–132. 10.1053/j.ackd.2017.10.011 29580576 PMC11000253

[3] Dunlay SM, Roger VL, Redfield MM. Epidemiology of heart failure with preserved ejection fraction. Nat Rev Cardiol 2017; 14: 591–602. 10.1038/nrcardio.2017.65 28492288

[4] Mosley JD, Levinson RT, Brittain EL, Gupta DK, Farber-Eger E, Shaffer CM, et al Clinical Features Associated With Nascent Left Ventricular Diastolic Dysfunction in a Population Aged 40 to 55 Years. Am J Cardiol 2018; S0002-9149: 30278–9. 10.1016/j.amjcard.2018.02.042 PMC5975107 29627106

[5] Nayor M, Enserro DM, Xanthakis V, Larson MG, Benjamin EJ, Aragam J, et al Comorbidities and Cardiometabolic Disease: Relationship With Longitudinal Changes in Diastolic Function. JACC Heart Fail 2018; 6: 317–325. 10.1016/j.jchf.2017.12.018 29525334 PMC5878123

[6] Reis JP, Allen NB, Bancks MP, Carr JJ, Lewis CE, Lima JA, et al Duration of Diabetes and Prediabetes During Adulthood and Subclinical Atherosclerosis and Cardiac Dysfunction in Middle Age: The CARDIA Study. Diabetes Care 2018; 41: 731–738. 10.2337/dc17-2233 29317451 PMC5860835

[7] Nagueh SF, Smiseth OA, Appleton CP, Byrd BF 3rd, Dokainish H, Edvardsen T, et al; Houston, Texas; Oslo, Norway; Phoenix, Arizona; Nashville, Tennessee; Hamilton, Ontario, Canada; Uppsala, Sweden; Ghent and Liège, Belgium; Cleveland, Ohio; Novara, Italy; Rochester, Minnesota; Bucharest, Romania; and St. Louis, Missouri. Recommendations for the Evaluation of Left Ventricular Diastolic Function by Echocardiography: An Update from the American Society of Echocardiography and the European Association of Cardiovascular Imaging. Eur Heart J Cardiovasc Imaging 2016; 17: 1321–1360. 10.1093/ehjci/jew082 27422899

[8] Mitter SS, Shah SJ, Thomas JD. A Test in Context: E/A and E/e’ to Assess Diastolic Dysfunction and LV Filling Pressure. J Am Coll Cardiol 2017; (69):1451–1464. 28302294 10.1016/j.jacc.2016.12.037

[9] Liberati A, Altman DG, Tetzlaff J, Mulrow C, Gøtzsche PC, Ioannidis JP, et al The PRISMA statement for reporting systematic reviews and meta-analyses of studies that evaluate health care interventions: explanation and elaboration. J Clin Epidemiol. 2009; 62: e1–34. 10.1016/j.jclinepi.2009.06.006 19631507

[10] Kerner W, Brückel J; German Diabetes Association. Definition, classification and diagnosis of diabetes mellitus. Exp Clin Endocrinol Diabetes. 2014; 122: 384–6. 10.1055/s-0034-1366278 25014088

[11] GA Wells, B Shea, D O’Connell, J Peterson, V Welch, M Losos, et al. The Newcastle-Ottawa Scale (NOS) for assessing the quality of nonrandomized studies in meta-analyses. http://www.ohri.ca/programs/clinical_epidemiology/oxford.htm (accessed 4 03 2018)

[12] Weir CJ, Butcher I, Assi V, Lewis SC, Murray GD, Langhorne P, et al Dealing with missing standard deviation and mean values in meta-analysis of continuous outcomes: a systematic review. BMC Med Res Methodol 2018; 18: 25–39. 10.1186/s12874-018-0483-0 29514597 PMC5842611

[13] Higgins JPT, Green S (editors). Cochrane Handbook for Systematic Reviews of Interventions Version 5.1.0 [updated March 2011]. The Cochrane Collaboration, 2011 http://handbook.cochrane.org.

[14] Shamseer L, Moher D, Clarke M, Ghersi D, Liberati A, Petticrew M, et al; PRISMA-P Group. Preferred reporting items for systematic review and meta-analysis protocols (PRISMA-P) 2015: elaboration and explanation. BMJ 2015; 350: g7647 10.1136/bmj.g7647 25555855

[15] Tayebjee MH, Lim HS, Nadar S, MacFadyen RJ, Lip GY. Tissue inhibitor of metalloproteinse-1 is a marker of diastolic dysfunction using tissue doppler in patients with type 2 diabetes and hypertension. Eur J Clin Invest 2005; 35: 8–12. 10.1111/j.1365-2362.2005.01438.x 15638813

[16] Govind SC, Brodin LA, Nowak J, Arvind SR, Ramesh SS, Netyö A, et al Microalbuminuria and left ventricular function in type 2 diabetes: a quantitative assessment by stress echocardiography in the Myocardial Doppler in Diabetes (MYDID) Study III. Scand Cardiovasc J 2007; 41: 363–9. 10.1080/14017430701604598 17924282

[17] Yazici M, Ozdemir K, Gonen MS, Kayrak M, Ulgen MS, Duzenli MA, et al Is there any relationship between metabolic parameters and left ventricular functions in type 2 diabetic patients without evident heart disease? Echocardiography 2008; 25: 675–82. 10.1111/j.1540-8175.2008.00690.x 18445056

[18] Mogelvang R, Sogaard P, Pedersen SA, Olsen NT, Schnohr P, Jensen JS. Tissue Doppler echocardiography in persons with hypertension, diabetes, or ischaemic heart disease: the Copenhagen City Heart Study. Eur Heart J 2009; 30: 731–9. 10.1093/eurheartj/ehn596 19176536

[19] Andersson C, Gislason GH, Weeke P, Hoffmann S, Hansen PR, Torp-Pedersen C, et al Diabetes is associated with impaired myocardial performance in patients without significant coronary artery disease. Cardiovasc Diabetol 2010; 9: 3 10.1186/1475-2840-9-3 20082690 PMC2818623

[20] Tayyareci Y, Yurdakul S, Tayyareci G, Nisanci Y, Umman B, Buğra Z. Impact of myocardial acceleration during isovolumic contraction in evaluating subclinical right ventricular systolic dysfunction in type 2 diabetes mellitus patients. Echocardiography 2010; 27: 1211–8 10.1111/j.1540-8175.2010.01237.x 20584066

[21] Ernande L, Bergerot C, Rietzschel ER, De Buyzere ML, Thibault H, Pignonblanc PG, et al Diastolic dysfunction in patients with type 2 diabetes mellitus: is it really the first marker of diabetic cardiomyopathy? J Am Soc Echocardiogr 2011; 24: 1268–1275. 10.1016/j.echo.2011.07.017 21907542

[22] Ceyhan K, Kadi H, Koç F, Celik A, Oztürk A, Onalan O. Longitudinal left ventricular function in normotensive prediabetics: a tissue Doppler and strain/strain rate echocardiography study. J Am Soc Echocardiogr 2012; 25: 349–56. 10.1016/j.echo.2011.11.018 22177116

[23] Çiftel S, Içağasıoğlu S, Yıldız G, Tekin G, Aydin H. Association of left ventricular diastolic dysfunction with elevated NT-proBNP in type 2 diabetes mellitus patients with preserved ejection fraction: the supplemantary role of tissue doppler imaging parameters and NT-proBNP levels. Diabetes Res Clin Pract 2012; 96: 179–86. 10.1016/j.diabres.2011.12.021 22240157

[24] Conte L, Fabiani I, Barletta V, Bianchi C, Maria CA, Cucco C, et al Early Detection of Left Ventricular Dysfunction in Diabetes Mellitus Patients with Normal Ejection Fraction, Stratified by BMI: A Preliminary Speckle Tracking Echocardiography Study. J Cardiovasc Echogr 2013; 23: 73–80. 10.4103/2211-4122.123953 28465889 PMC5353391

[25] Erdogan D, Yucel H, Uysal BA, Ersoy IH, Icli A, Akcay S, et al Effects of prediabetes and diabetes on left ventricular and coronary microvascular functions. Metabolism 2013; 62: 1123–30. 10.1016/j.metabol.2013.02.011 23557591

[26] Atas H, Kepez A, Atas DB, Kanar BG, Dervisova R, Kivrak T, et al Effects of diabetes mellitus on left atrial volume and functions in normotensive patients without symptomatic cardiovascular disease. J Diabetes Complications 2014; 28: 858–62. 10.1016/j.jdiacomp.2014.07.010 25130919

[27] Bakirci EM, Demirtas L, Degirmenci H, Topcu S, Demirelli S, Hamur H, et al Relationship of the total atrial conduction time to subclinical atherosclerosis, inflammation and echocardiographic parameters in patients with type 2 diabetes mellitus. Clinics 2015; 70: 73–80. 10.6061/clinics/2015(02)01 25789513 PMC4351316

[28] Loncarevic B, Trifunovic D, Soldatovic I, Vujisic-Tesic B. Silent diabetic cardiomyopathy in everyday practice: a clinical and echocardiographic study. BMC Cardiovasc Disord 2016; 16: 242 10.1186/s12872-016-0395-z 27894255 PMC5126872

[29] Vukomanovic V, Tadic M, Suzic-Lazic J, Kocijancic V, Celic V. The relationship between heart rate variability and left ventricular layer-specific deformation in uncomplicated diabetic patients. Int J Cardiovasc Imaging 2017; 33: 481–490. 10.1007/s10554-016-1023-9 27853970

[30] Marwick TH, Ritchie R, Shaw JE, Kaye D. Implications of Underlying Mechanisms for the Recognition and Management of Diabetic Cardiomyopathy. J Am Coll Cardiol 2018; 71: 339–351. 10.1016/j.jacc.2017.11.019 29348027

[31] Nichols GA, Hillier TA, Erbey JR, Brown JB. Congestive heart failure in type 2 diabetes: prevalence, incidence, and risk factors. Diabetes Care 2001; 24: 1614–9. 11522708 10.2337/diacare.24.9.1614

[32] Echouffo-Tcheugui JB, Xu H, DeVore AD, Schulte PJ, Butler J, Yancy CW, et al Temporal trends and factors associated with diabetes mellitus among patients hospitalized with heart failure: Findings from Get With The Guidelines-Heart Failure registry. Am Heart J 2016; 182: 9–20. 10.1016/j.ahj.2016.07.025 27914505

[33] MacDonald MR, Petrie MC, Varyani F, Ostergren J, Michelson EL, Young JB, et al; CHARM Investigators. Recognition and Management of Diabetic Cardiomyopathy. J Am Coll Cardiol 2018; 71: 339–351. Impact of diabetes on outcomes in patients with low and preserved ejection fraction heart failure: an analysis of the Candesartan in Heart failure: Assessment of Reduction in Mortality and morbidity (CHARM) programme. Eur Heart J 2008; 29: 1377–85. 10.1016/j.jacc.2017.11.019 18413309

[34] Seferović PM, Paulus WJ. Clinical diabetic cardiomyopathy: a two-faced disease with restrictive and dilated phenotypes. Eur Heart J 2015; 36: 1718–27, 1727a–1727c. 10.1093/eurheartj/ehv134 25888006

[35] Poornima IG, Parikh P, Shannon RP. Diabetic cardiomyopathy: the search for a unifying hypothesis. Circ Res 2006; 98: 596–605. 10.1161/01.RES.0000207406.94146.c2 16543510

[36] Boyer JK, Thanigaraj S, Schechtman KB, Pérez JE. Prevalence of ventricular diastolic dysfunction in asymptomatic, normotensive patients with diabetes mellitus. Am J Cardiol 2004; 93: 870–5. 10.1016/j.amjcard.2003.12.026 15050491

[37] Liu JE, Palmieri V, Roman MJ, Bella JN, Fabsitz R, Howard BV, et al The impact of diabetes on left ventricular filling pattern in normotensive and hypertensive adults: the Strong Heart Study. J Am Coll Cardiol 2001; 37: 1943–9. 11401136 10.1016/s0735-1097(01)01230-x

[38] From AM, Scott CG, Chen HH. The development of heart failure in patients with diabetes mellitus and pre-clinical diastolic dysfunction a population-based study. J Am Coll Cardiol 2010; 55: 300–5. 10.1016/j.jacc.2009.12.003 20117433 PMC3878075

[39] Redfield MM, Jacobsen SJ, Burnett JC Jr, Mahoney DW, Bailey KR, Rodeheffer RJ. Burden of systolic and diastolic ventricular dysfunction in the community: appreciating the scope of the heart failure epidemic. JAMA 2003; 289: 194–202. 12517230 10.1001/jama.289.2.194

[40] Halley CM, Houghtaling PL, Khalil MK, Thomas JD, Jaber WA. Mortality rate in patients with diastolic dysfunction and normal systolic function. Arch Intern Med 2011; 171: 1082–7. 10.1001/archinternmed.2011.244 21709107

[41] Kane GC, Karon BL, Mahoney DW, Redfield MM, Roger VL, Burnett JC Jr, et al Progression of left ventricular diastolic dysfunction and risk of heart failure. JAMA 2011; 306: 856–63. 10.1001/jama.2011.1201 21862747 PMC3269764

[42] Yancy CW, Jessup M, Bozkurt B, Butler J, Casey DE Jr, Colvin MM, et al 2017 ACC/AHA/HFSA Focused Update of the 2013 ACCF/AHA Guideline for the Management of Heart Failure: A Report of the American College of Cardiology/American Heart Association Task Force on Clinical Practice Guidelines and the Heart Failure Society of America. J Am Coll Cardiol 2017; 70: 776–803. 10.1016/j.jacc.2017.04.025 28461007

