# Supplementary material for: The E/e’ ratio difference between subjects with type 2 diabetes and controls. A meta-analysis of clinical studies
Source: PLoS One. 2018 Dec 27;13(12):e0209794. doi: 10.1371/journal.pone.0209794 (PMC6307698; doi:10.1371/journal.pone.0209794)
Supplement: S2 Table — Subgroups/Sensitivity analyses of the overall effect of studies with ≤ 45 and > 45 participants; studies with NOS > 6 and ≥ 6 score. (DOCX) [file pone.0209794.s002.docx]

S2 Table. Supplementary. Subgroups/Sensitivity analyses of the overall effect of studies with ≤ 45 and > 45 participants; studies with NOS > 6 and ≥ 6 score.

|  | Overall effect  WMD (95% CI) | Z | P | I^2^ | p |  |
| --- | --- | --- | --- | --- | --- | --- |
| Participants |  |  |  |  |  |  |
| ≤ 45 (6 studies) | 1.61 (0.51, 2.72) | 2.85 | 0.004 | 83.5 % | < 0.001 |  |
| > 45 (9 studies) | 2.27 (1.42, 3.11) | 5.26 | < 0.001 | 91.8 % | < 0.001 |  |
| NOS |  |  |  |  |  |  |
| < 6 (4 studies) | 1.29 (-0.14, 2.72) | 1.76 | 0.078 | 85.8 % | < 0.001 |  |
| ≥ 6 (11 studies) | 2.28 (1.55, 3.02) | 6.08 | < 0.001 | 89.9 % | < 0.001 |  |

WMD: weight mean difference.
