# Supplementary material for: The E/e’ ratio difference between subjects with type 2 diabetes and controls. A meta-analysis of clinical studies
Source: PLoS One. 2018 Dec 27;13(12):e0209794. doi: 10.1371/journal.pone.0209794 (PMC6307698; doi:10.1371/journal.pone.0209794)
Supplement: S3 Table — (DOCX) [file pone.0209794.s003.docx]

S3 Table. One Newcastle-Ottawa quality Assessment Scale

(NOS).

| **Author (Year)** | **Selection** | **Comparability** | **Exposure/Outcome** |
| --- | --- | --- | --- |
| Tayebjee (2005) | ✶✶✶✶ | ✶✶ | ✶ |
| Govind (2007) | ✶✶✶ | ✶ | ✶ |
| Yazici (2008) | ✶✶✶✶ | ✶ | ✶ |
| Mogelvang (2009) | ✶✶✶ | ✶ | ✶✶ |
| Andersson (2010) | ✶✶✶✶ | ✶✶ | ✶✶ |
| Tayyareci (2010) | ✶✶✶✶ | ✶✶ | ✶ |
| Ernande (2011) | ✶✶✶✶ | ✶✶ | ✶ |
| Ceyhan (2012) | ✶✶✶✶ | ✶✶ | ✶✶ |
| çiftel (2012) | ✶✶✶ | ✶ | ✶ |
| Conte (2013) | ✶✶ | ✶ | ✶ |
| Erdogan (2013) | ✶✶✶ | ✶ | ✶ |
| Atas (2014) | ✶✶✶✶ | ✶✶ | ✶ |
| Bakirci (2015) | ✶✶✶✶ | ✶✶ | ✶ |
| Loncarevic (2016) | ✶✶✶ | ✶✶ | ✶✶ |
| Vukomovic (2017) | ✶✶ | ✶ | ✶ |
