# Supplementary material for: The E/e’ ratio difference between subjects with type 2 diabetes and controls. A meta-analysis of clinical studies
Source: PLoS One. 2018 Dec 27;13(12):e0209794. doi: 10.1371/journal.pone.0209794 (PMC6307698; doi:10.1371/journal.pone.0209794)
Supplement: S4 Table — (PDF) [file pone.0209794.s004.pdf]

	Selection	Comparability	Exposure or Outcome
Andersson 2010	+	+	+
Atas 2014	+	+	?
Bakirci 2015	+	+	?
Ceyhan 2012	+	+	+
çiftel 2012	?	?	?
Conte 2013	+	?	?
Erdogan 2013	?	?	?
Ernande 2011	+	+	?
Govind 2007	?	?	?
Loncarevic 2016	?	+	+
Mogelvang 2009	?	?	+
Tayebjee 2005	+	+	?
Tayyareci 2010	+	+	?
Vukomovic 2017	?	?	?
Yazici 2008	+	?	?
