# Supplementary figures and images for: The E/e’ ratio difference between subjects with type 2 diabetes and controls. A meta-analysis of clinical studies
Source: PLoS One. 2018 Dec 27;13(12):e0209794. doi: 10.1371/journal.pone.0209794 (PMC6307698; doi:10.1371/journal.pone.0209794)

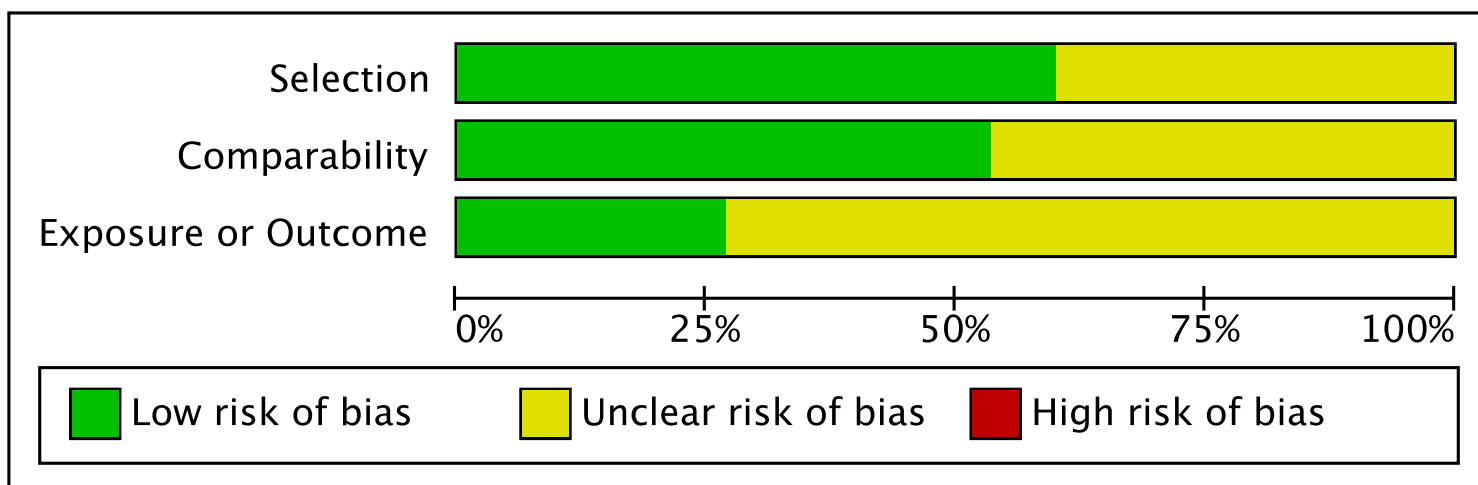

Supplement: S5 Table — (PDF) [file pone.0209794.s005.pdf]
